# Writing for publication in peer reviewed Nursing journals - the need to consider the Global Audience

**DOI:** 10.1590/1518-8345.0000.3906

**Published:** 2023-03-27

**Authors:** Fiona Timmins

**Affiliations:** 1 University College Dublin, School of Nursing, Midwifery and Health Systems, Dublin, Ireland.


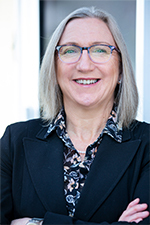
 The aim of this editorial is to provide a brief overview of advice when preparing manuscripts for publication in peer reviewed nursing journals. In particular, I would like to highlight the need for authors to write for a global audience. Writing for publication can be challenging and rejection is common. As a great majority of scientific publication is through the medium of English, this creates a difficulty for those for whom this is a second language^1^. People also struggle with academic writing skills. However, it is my view that the greatest omission is that authors often fail to consider the requirements of writing for a global audience. 

The aims and scope of most Journals will address the need for novelty and internationality. As such careful preparation is needed to ensure that the manuscript meets these key criteria. This preparation involves an up-to-date search of the topic within the literature generally, but also specifically within the target Journal. By exploring the literature anew, you can begin to re-examine the unique contribution of your study and consider a strong argument that you can build into the paper that identifies its unique contribution to knowledge. It is important to remember here that you are aiming to contribute to knowledge globally. If your study has had a local, regional, or even national contribution, this may not be sufficient. You will need to consider this contribution and how it now further contributes to the global agenda in the field. As Thistlethwaite & Anderson^2^ point out: “work should not be country-centric without reference to a global perspective”.

Moreover, it will be very important to explain and outline the context within which your research took place. This will be important to inform readers of why the research had such fundamental importance. Nursing research is evolving at a different pace across the globe. What might seem quite novel in one domain may not be in another. As such explaining the context importance and relevance of the study in the host area can provide important insights that make your work more likely to be published. 

It is also important to remember the structure of the manuscript beyond the basic formatting. One common error is that the study is presented in the manuscript in its most basic form. That is, a short background (often with out-of-date literature because the work began some time back), a basic outline of the study and a repeat of the findings in the discussion, with little evidence of novelty or contribution to the body of knowledge. Rather than this, the manuscript ought to be considered as an argument. At the outset what is known about a topic is presented, leading to what is unknown and important to find out, and leading to a clear justification for your research. The findings out to comprise key and important findings, and the most important of these are drawn out in the discussion. At that point of the argument (the discussion), it is important not to simply repeat the findings, but to identify how the selected important findings contribute to the body of knowledge in the field globally. This may translate into implications for research, education, and practice, with a clear indication of where the limitations lie. 

How you approach the discussion is vital. Those reading your manuscript, who in the first instance will be the Editor or reviewer, will be interested to know the main contribution of your manuscript, particularly in relation to novelty. They will be concerned with what key message are you trying to convey to a global audience and will likely ask the “so what”? question^2^. Indeed, some of the key reasons why manuscripts are rejected relate to out-of-date literature, lack of novelty and failure to make a point^3^. Editors are looking for meaningful and impactful research, therefore it is important to express the key novel findings and their implications globally.

In short, while your work has already had clear success in its original context and for its original purpose, it now needs to be extensively reworked, beyond simply formatting, to fit with the target journal. This preparation takes quite a considerable amount of time and be prepared to set aside at least 60- 80 hours for this task. Updating your literature and considering anew the contribution to knowledge of the work and preparing a clear argument within the manuscript will help you towards success. Remember that “manuscripts that repeat old studies without producing any reasonable new information and have been replicated without justification really don’t appeal to us (Editors)”^4^. The most important parts of the paper are the background/introduction and discussion, wherein the key arguments, related to the study’s novelty and contribution can be articulated. This ‘new’ manuscript needs to clearly meet the aims and scope of the target journal, provide a novel perspective on the topic, and speak to a global audience. Spending time on this preparation, rather than simply a rushed edit will reap rewards in terms of your success in publishing for the future.
